# A framework to guide storytelling as a knowledge translation intervention for health-promoting behaviour change

**DOI:** 10.1186/s43058-022-00282-6

**Published:** 2022-03-28

**Authors:** Stephanie P. Brooks, Gabrielle L. Zimmermann, Michael Lang, Shannon D. Scott, Denise Thomson, Gil Wilkes, Lisa Hartling

**Affiliations:** 1 Alberta SPOR SUPPORT Unit – Learning Health System Team, Department of Medicine, University of Alberta, Edmonton, Canada; 2 Department of Community Health Sciences, Cumming School of Medicine, University of Calgary, Calgary, Canada; 3 Faculty of Nursing, University of Calgary, Calgary, Canada; 4 Faculty of Nursing, University of Alberta, Edmonton, Canada; 5 Information Design, School of Communication Studies, Mount Royal University, Calgary, Canada; 6 Alberta Research Centre for Health Evidence, Department of Pediatrics, University of Alberta, Edmonton, Canada

**Keywords:** Knowledge translation, Storytelling, Framework, Behaviour change, Health promotion

## Abstract

**Background:**

Stories can be a powerful tool to increase uptake of health information, a key goal of knowledge translation (KT). Systematic reviews demonstrate that storytelling (i.e. sharing stories) can be effective in changing health-promoting behaviours. Though an attractive KT strategy, storytelling is a complex approach requiring careful planning and consideration of multiple factors. We sought to develop a framework to assist KT researchers and practitioners in health contexts to consider and develop effective KT interventions that include stories or storytelling.

**Methods:**

We conducted a broad search of the literature to identify studies that used storytelling as a KT intervention across different disciplines: health research, education, policy development, anthropology, organizational development, technology research, and media. We extracted purposes, theories, models, mechanisms, and outcomes and then mapped the theoretical and practical considerations from the literature onto the Medical Research Council guidance for complex interventions. The theoretical and practical considerations uncovered comprised the basis of the storytelling framework development. Through discussion and consensus, methodological experts refined and revised the framework for completeness, accuracy, nuance, and usability.

**Results:**

We used a complex intervention lens paired with existing behaviour change techniques to guide appropriate theory-based intervention planning and practical choices. An intentional approach to the development of story-based KT interventions should involve three phases. The theory phase specifies the goal of the intervention, mechanisms of action, and behaviour change techniques that will achieve the intended effects. The modelling phase involves development and testing using an iterative approach, multiple methods and engagement of end-users. Finally, formal evaluation using multiple methods helps determine whether the intervention is having its intended effects and value added.

**Conclusions:**

This framework provides practical guidance for designing story-based KT interventions. The framework was designed to make explicit the requisite considerations when determining the appropriateness and/or feasibility of storytelling KT, clarify intervention goals and audience, and subsequently, support the development and testing of storytelling interventions. The framework presents considerations as opposed to being prescriptive. The framework also offers an opportunity to further develop theory and the KT community’s understanding of effectiveness and mechanisms of action in storytelling interventions.

**Supplementary Information:**

The online version contains supplementary material available at 10.1186/s43058-022-00282-6.

Contributions to the literature

- Stories (i.e. narratives of patients, friends, family, and caregiver experiences) are a tremendously popular and effective medium to support the uptake of research evidence. However, literature on interventions that include stories for knowledge translation rarely reports how stories are developed, the theory that underlies these interventions, or which storytelling approaches work or not in which contexts.
- This trend creates both methodological and scientific knowledge gaps for successful story-based intervention development.
- This paper provides a theory-based framework to help intervention developers plan, develop, evaluate, and report on story-based interventions, in turn helping the research community to build a more robust science of storytelling.

## Introduction

Stories are ubiquitous and have been used for communication and entertainment for thousands of years. Stories engage us by evoking emotion and can compel us to think or behave differently. Indeed, stories are available as communication tools across disciplines and can stem from numerous, and oftentimes competing, theoretical perspectives [1]. While the breadth of story development approaches makes traditional measurement and quantification difficult [2, 3], stories can be a useful tool for disseminating and communicating evidence (i.e. knowledge translation or “KT”) in healthcare [4–6]. Furthermore, recent systematic reviews have found that changing health-promoting behaviours using storytelling (i.e. sharing stories) appears to be promising, as stories help people identify with another, picture themselves behaving differently, and in turn, reduce resistance and inspire new health behaviours [6, 7]. However, there is little guidance to help determine how and when to use stories or storytelling as a KT intervention [6, 7]. Given the observations and limitations of the extant literature, there are still several research gaps including addressing contexts where stories are most appropriate, the theoretical mechanisms through which stories influence behaviour, and how to format and present stories effectively [6, 7]. Nevertheless, the existing literature holds valuable insight into the considerations required, and possible avenues to take, when designing KT interventions that include storytelling or stories.

Storytelling is an attractive KT strategy but is a complex approach that, to be high quality and successful, requires thoughtful planning and full consideration of multiple components. In this article, we present a step-by-step framework designed to assist KT researchers and practitioners in health contexts who are considering KT interventions that include storytelling or stories. A priori, we did not situate ourselves in a given perspective or theory. We set out to explore how stories have been used by others and let the findings of those reports guide the framework development. The resulting framework was designed to make explicit the requisite considerations when determining the appropriateness and/or feasibility of storytelling KT, clarify intervention goals and audience, and subsequently, to support the development, testing, and evaluation of KT interventions.

The intention of this framework is to guide health-promoting behaviour change interventions that use stories in the context of KT. That is to say, the framework supports work to deliver messages derived from existing health evidence, as opposed to other story-based methodologies that employ storytelling for different ends (i.e. research, therapeutic value, patient engagement, and healthcare quality improvement). As such, when we refer to stories in this framework, we are referring to scripts written by health researchers in collaboration with end-user groups to deliver or complement evidence-based messages. When we use the term storytelling in the framework, we are referring to the written script as well as all the potential story delivery mediums (e.g. storybook, video, and audio recording), that together encompass the intervention design.

## Methods for framework development

A core group of KT experts (SB, GZ, DT, LH) performed the foundational work required to build the first iteration of the framework. We scanned the literature to identify a breadth of studies that used storytelling as a KT intervention across many different disciplines. We collected literature in health research, education, policy development, anthropology, organizational development, technology research, and media studies to examine various experiences using storytelling as a complex intervention (See Additional file 1). We then extracted purposes, theories, models, mechanisms and outcomes from the articles and mapped the theoretical and practical considerations pulled from the literature onto the Medical Research Council (MRC) guidance for complex interventions [8].

Reviewing the theories, models, and mechanisms reported in the literature uncovered a wide breadth of approaches towards stories, both theoretically and methodologically. Our review also revealed similarities to the Behaviour Change Wheel, including constructs from the Capability, Opportunity, Motivation to Behaviour model [9], the Theoretical Domains Framework (TDF), and behaviour change techniques. Given the breadth of theories guiding different storytelling interventions, we found TDF particularly useful as it was designed, “to simplify and integrate a plethora of behaviour change theories and make theory more accessible to, and usable by, other disciplines” [10].

The theoretical and practical considerations uncovered comprised the basis of our storytelling framework development. Additional methodological experts (SS, GW, ML) helped refine and revise the drafted framework based on their expertise using stories in KT activities and interventions. The additional experts helped to assess and deepen the completeness, accuracy, nuance, and usability of the storytelling framework.

## Results: A framework to guide complex storytelling interventions

This section walks through the step-by-step guidance (i.e. the storytelling framework) developed from existing literature to assist researchers in developing, evaluating, and reporting theory-based knowledge translation interventions that incorporate stories. Specifically, the framework is modelled from the Medical Research Council guidance on developing and evaluating complex interventions (MRC Framework) [8], which helps to guide intervention development from theory-based design, modelling, and evaluation. The guidance presented expands on how stories fit into established theories and frameworks and provides insight into required intervention development considerations across design, modelling, and evaluation phases. Additionally, here we include illustrative examples at each step pulled from the literature as well as from experiences of the authorship team.

The literature in our scan highlighted the inconsistencies of reporting storytelling intervention design and outcomes, which helps to explain the lack of concrete storytelling guidance [6, 7]. One potential reason for the paucity of guidance for storytelling in KT is that stories are often one component of multi-component interventions. Similarly, multiple mechanisms are at work simultaneously when developing, telling and receiving stories (e.g. beliefs, knowledge, social elements [11]). Stories used in health-promoting behaviour change interventions will also be delivered and received in a variety of decision-making contexts. Decisions may be made alone, with a health care provider, with health care teams, and may involve multiple family members [12]. Finally, messages can exist on a spectrum from clear with simple actions (e.g. graphic storyboards with explicit messaging) to complex and ambiguous (e.g. a research-based dance performed to elicit questions, conversation, or question assumptions [11–13]). Indeed, there is an art to developing stories that resonate with target populations in different contexts while maintaining evidence-based health messaging [14, 15]. The way in which context interacts with the intervention itself adds to the complexity of intervention development in storytelling, highlighting the importance of feasibility and acceptability testing before intervention implementation.

**Fig. 1:**
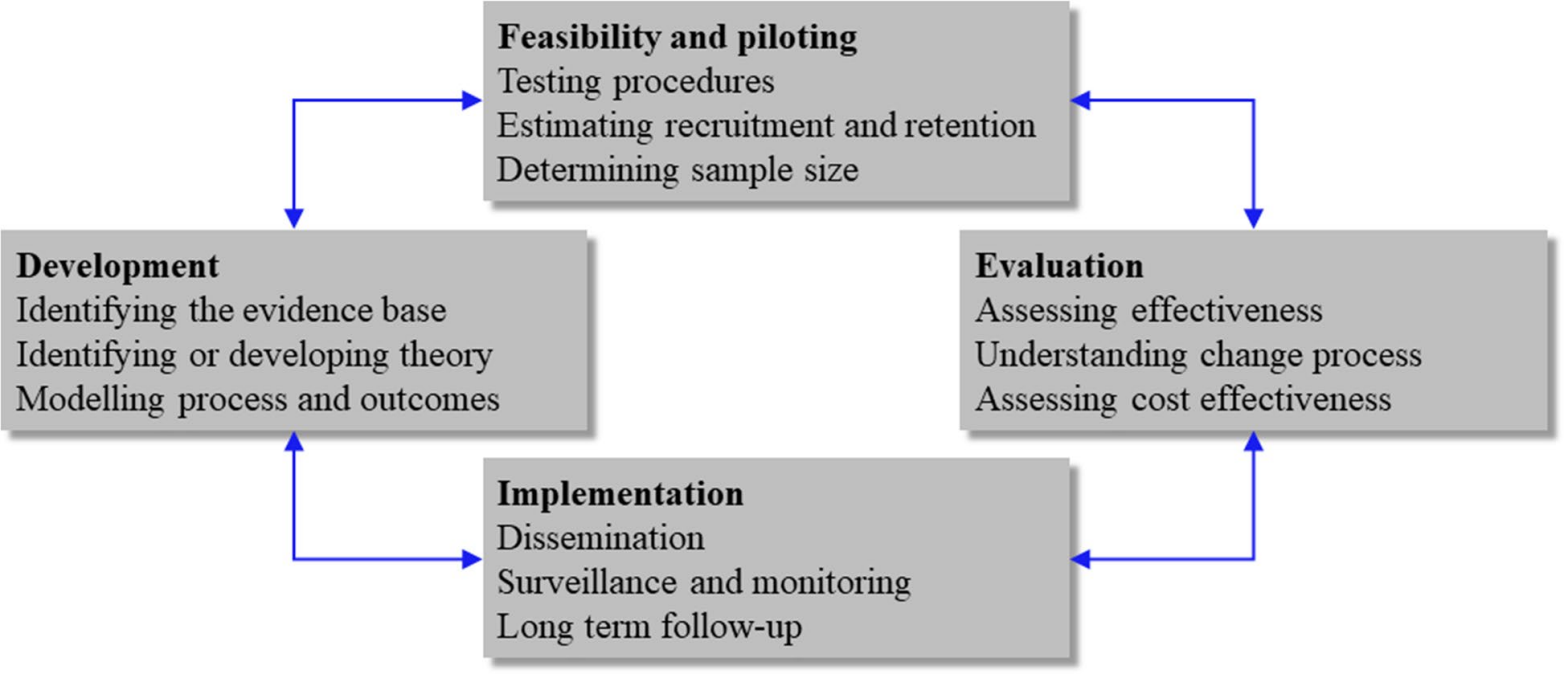
Medical Research Council Framework for developing and evaluating complex interventions. Reprinted from Craig et al. (2008) [16]; permission to reprint was granted by BMJ Publishing Group Ltd

Given these factors, it is helpful to consider storytelling as a complex intervention, especially when behaviour change is the desired outcome. Complex interventions contain several interacting components and dimensions of complexity such as number of target groups, variability of outcomes, and degree of fidelity required/tailoring allowed [8]. The MRC Framework assists researchers in developing, investigating feasibility, and evaluating the effectiveness and outcomes of such interventions (Fig. 1 [16];). The MRC Framework was designed to help teams develop tailorable interventions that meet localized or contextual needs and have measurable outcomes. As guidance to identify appropriate intervention design and evaluation methods, the MRC Framework can be valuable for thinking about the appropriateness of storytelling methods and how to measure their impact.

**Fig. 2:**
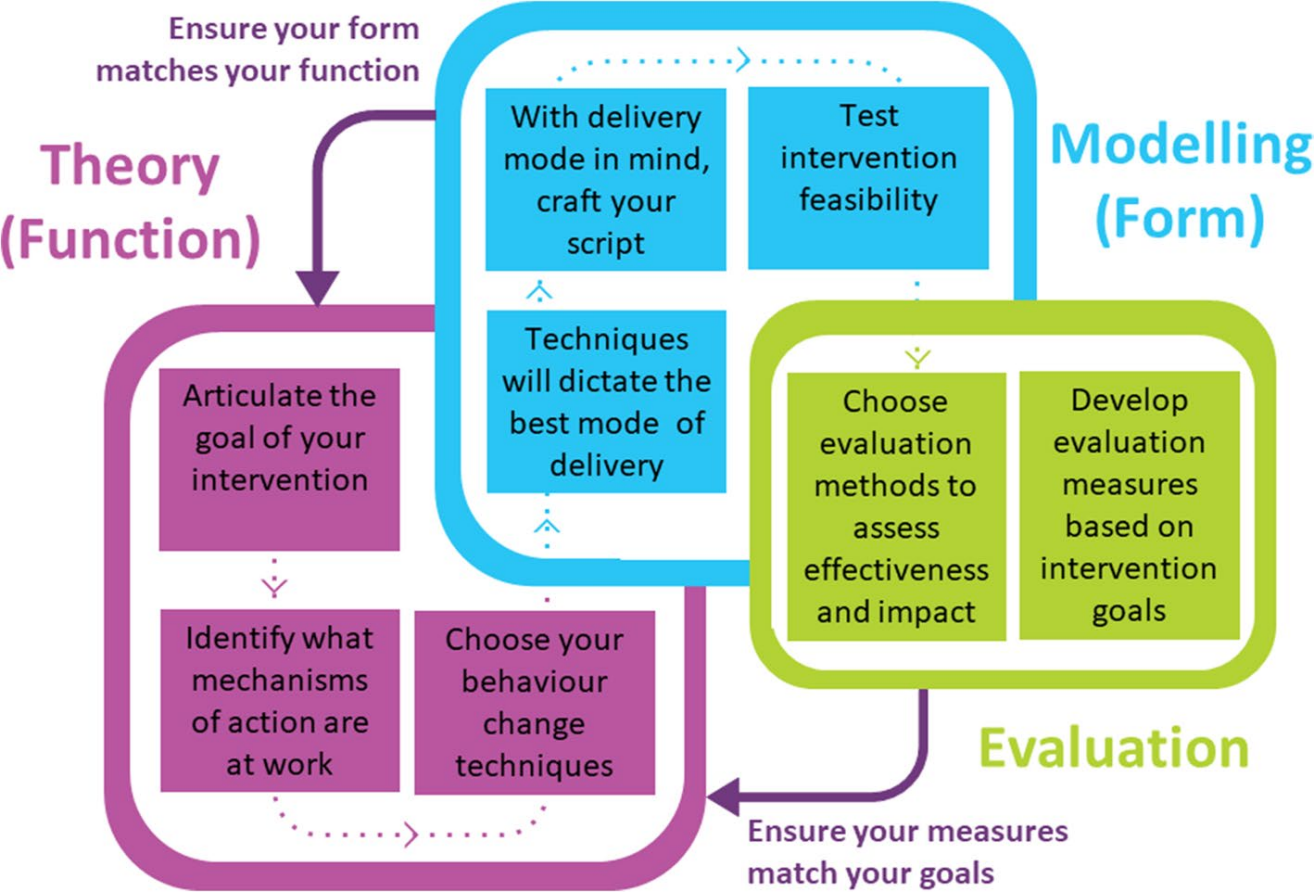
Developing a storytelling intervention

We modified the MRC Framework, which recommends iteratively developing, testing and evaluating an intervention before implementing it in a given setting, to highlight how it applies to storytelling as a KT intervention (Fig. 2). For storytelling interventions, it is important to consider how following the MRC Framework’s theory-modelling-evaluation phases can help craft the intervention. The development of stories themselves requires a significant amount of effort and resources. Moreover, if stories are to be the foundation of a successful intervention, they need to be engaging and compelling and effectively present the health information. Following a sequential process of storytelling intervention design, in alignment with the MRC framework, can help reduce design uncertainties and keep the focus on the behaviour change goals. It is helpful to think of the theory and evaluation phases as the science of storytelling, while the modelling (i.e. development of script and delivery medium) is the art. Below we outline the processes of the theory, modelling and evaluation phases to help those building storytelling KT interventions understand the considerations that maintain the integrity of the evidence-based messaging while crafting compelling stories capable of changing behaviours.

### Theory phase

The theory phase establishes the function of the intervention, that is the means by which an intervention can change behaviour. Theory is essential in identifying what to target (behavioural determinants) and how to do this (techniques to change these determinants). During the theory phase, key early tasks include (1) clearly articulating the goal(s) of the intervention and (2) developing a theoretical understanding of the likely process of change by drawing on existing evidence and theory.

#### Considerations for KT goals

Clear articulation of the knowledge translation goal is required to identify if storytelling is truly the best/most appropriate method to use for an intervention. Furthermore, the goal will identify which storytelling methodology should be employed given the target population characteristics [17]. Those designing story-based interventions must consider the fundamental purpose as well as a number of internal and external influences that both the story’s content and the storytelling medium must address. Internal influences include existing audience beliefs and whether the audience is already aware and/or ready to act on the health issue of interest [18]. External influences include items such as the ethos of early compared to late adopters [19], the political environment, professional norms of the target audience, and timing of storytelling distribution will impact the appropriateness of intervention design for a given context [20]. Because stories will be presented in specific contexts, attention must be paid to the needs of the audience. For example, in vaccination promotion, stories will have to be crafted and shared differently depending on the target audience’s attitudes towards vaccines, e.g. acceptance, hesitancy, or refusal.

#### Identifying mechanisms of actions and corresponding behaviour change techniques (i.e. process/theory of change)

After articulating the goal(s) of the storytelling intervention, teams must understand the determinant(s) affecting the behaviour change being targeted (i.e. the barriers to change) and identify which mechanisms of action will create the desired behaviour change. Mechanisms of action are defined as the processes through which an intervention affects behaviour, with behaviour being anything a person does in response to internal or external events. Subsequently, teams should identify possible behaviour change techniques (BCTs) that are the active components designed to change behaviour. There are numerous possible mechanisms of action and even more BCTs to employ in healthcare contexts [21, 22]; therefore, these steps require significant dedicated time and thought. Recognizing the importance and the difficulty of these steps, Michie and colleagues [21, 22] provide extensive and useful guidance for identifying mechanisms of action and corresponding BCTs. First, they constructed an evolving taxonomy of BCTs to be used for developing theory-based behaviour change interventions. Second, they built the Behaviour Change Wheel (BCW) to help identify mechanisms and existing theory of change upon which to base subsequent design decisions [9]. At its heart is the Capability, Opportunity, Motivation to Behaviour (COM-B) model of behaviour. The COM-B model posits that people cannot and/or will not change their behaviour without a sufficient combination of capability, opportunity, and motivation to change. Thus, health-promoting storytelling interventions need to respond to people’s behaviours while keeping in mind that an imbalance of these components may hinder change. The COM-B model outlines sources of behaviour that may need to change and links to nine intervention functions in the BCW which are aimed at addressing deficits in one or more of the three central components [9].

Intervention functions and associated BCTs are the broad categories and specific techniques, respectively, that can help identified individuals to change their behaviours in ways desired by the intervention. While the literature we reviewed did not specifically identify BCTs or intervention functions, several did mention mechanisms. Depending on the level of detail provided, many of the mechanisms we identified in the papers we reviewed could be coded/mapped as behavioural determinants (such as those outlined in the Theoretical Domains Framework [TDF]) [23], intervention functions (found in the BCW) or behaviour change techniques [9, 21, 22, 24]. Table 1 shows this mapping to provide a reference of the different approaches already detailed in the literature and to illustrate the utility of using frameworks like TDF and BCW to help conceptualize theory-based storytelling interventions. However, researchers need to be more explicit in their descriptions of how they expect storytelling to have the intended effect so that the mechanisms can be evaluated more appropriately (see Table 2 for examples).

**Table 1:** Mapping mechanisms to theoretical domain framework (TDF) domains, intervention functions or behaviour change techniques (BCTs) [21–23]

Mechanisms from storytelling articles (bolded = main categories)	TDF domain, intervention function (IF) and/or BCT
**Relational (social) strategies**	
Appreciate other perspectives	**BCT** – Framing/reframing
Developing trust	**TDF** – Social influences
Importance of relationships	**TDF** – Social influences
Share personal understanding with others	**TDF** – Social influences
Cultural embeddedness	**TDF** – Social influences
**Communication strategies**	
Animation with embedded script (explore difficult issues in non-threatening form)	**TDF** – Emotion
Breaking down misconceptions, perceptions, and confusion that can inhibit knowledge interpretation	**TDF** – Knowledge
	**IF** – Education
Entertainment-education (intentional placement of educational content in entertainment messages)	**IF** – Modelling
Make abstract or conceptual content more understandable	**TDF** – Knowledge
	**IF** – Education
Multi-media in teaching helps with knowledge retention and comprehension	**TDF** – Memory, attention, and decision process
Persuasion (through a compelling story)	**IF** – Persuasion
**Mental strategies**	
Empathic connection to story characters	**TDF** – Emotion
Identification/mirroring/homophily	**TDF** – Social role and identity
	**BCT** – Social comparison
Transactional relationship (relating stories to own life experience)	**BCT** – Social comparison
Persuasion (through memory, evaluation, dual-process controller)	**TDF** – Memory, attention, and decision process
Problem solving	**BCT** – Problem solving
Changing stereotypes to influence decision-making and choice	**TDF** – Social influences **BCT** – Framing/reframing
Motivation to learn and take action	**TDF** – Intentions
	**BCT** – Action planning
Increased literacy	**IF** – Education

**Table 2:** Examples of reporting intended change

	Smoking cessation intervention	Managing childhood illness intervention
**Goal**	Stop Smoking	Effectively manage various childhood illnesses [11, 22, 23] (i.e. know when and how to manage illness at home and when to seek medical or emergency care)
**Behavioural determinant(s)**	● Lack of knowledge of health effects of smoking (TDF) which is linked to capability (COM-B) ● Beliefs about consequences of smoking (TDF) which is linked to motivation (COM-B)	● Lack of knowledge around common childhood illnesses (TDF) ● Feeling alone in managing child health (TDF – emotions)
**Possible intervention functions**	● Education - if the primary function of the story is to increase knowledge ● Persuasion and/or modelling – if the function is to address beliefs about consequences	● Education – if function is to increase knowledge and confidence ● Modelling – if the function is to evoke emotion while sharing evidence-based messages
**Focus of the story**	Health consequences of smoking versus quitting smoking	Parents identifying an illness and deciding whether to seek out care or treat from home

Based on the goal of storytelling and the determinants of behaviour, the bottom-line function(s) of the intervention and related behaviour change techniques can be identified and used to guide the change mechanism. In Table 3, we have compiled a list of the most common intervention functions, identified from our scan of the literature. To aid in the identification of BCTs, there is an interactive Theory & Techniques Tool [25] based on a recent expert consensus study [26], which provides information about links between BCTs and their mechanisms of action. When selecting which BCTs to use, it is helpful to consider the APEASE criteria (affordability, practicality, effectiveness/cost-effectiveness, acceptability, side-effects/safety, equity) in the context of storytelling. Modelling the intervention with APEASE in mind will help choose BCTs that suit the intervention goal(s) and fit the context within which the intervention will be implemented [27]. Taking all of these elements into consideration can help determine the functions of a storytelling KT intervention which in turn informs the development and delivery of the story in the most relevant way and helps in the selection of appropriate indicators and outcome measurements to use when conducting feasibility testing and evaluation.

**Table 3:** Key intervention functions identified in the papers reviewed, linked to behaviour change techniques (BCTs) [21, 22]

Intervention function	Definition	Examples of frequently used BCTs
Education	Increasing understanding or knowledge	Information about health consequences Information about social consequences Prompts/cues
Modelling	Providing an example for people to aspire to or imitate	Demonstration of the behaviour
Persuasion	Using communication to induce positive or negative feelings or stimulate action	Credible source Information about health consequences Information about social consequences
Training	Imparting skills	Demonstration of the behaviour Instruction on how to perform a behaviour
Enablement	Increasing means/reducing barriers to increase capability (beyond education and training) or opportunity (beyond environmental restructuring)	Social support Problem solving Action planning

### Modelling phase

The crux of storytelling KT interventions exists in the modelling phase: how to build and deliver a compelling story that can inspire behaviour change while maintaining and highlighting an evidence-based message. After establishing the function of the intervention and the science underlying the behaviour change techniques, the modelling phase involves writing the story script and selecting the storytelling modality. This phase requires artistic ability to apply the science to the particular contexts in which the intervention will be implemented.

The MRC Framework suggests modelling the intervention before any real-world testing to help identify contextual factors that may influence the intended function and outcomes of the planned BCTs. Modelling storytelling interventions helps practitioners consider how to best develop messages and delivery methods to meet the needs of the message user. Teams can aim to reach a variety of potential users with their messaging (e.g. individual patients, health care practitioners, caregivers or other service providers working with target users, and health care decisions makers); thus, it is important to tailor the script to effectively reach and resonate with the intended user. A common approach to building stories is to employ stakeholder-engaged qualitative methods in which members of the intended audience will come together in interviews [3, 13, 15, 28–32], focus groups [33–36], or story development workshops [34, 37] with researchers to develop a story together. Involving stakeholders in message development is ideal for crafting scripts that resonate with the intended audience; however, researchers need to consider ethical challenges spanning the course of the storytelling project from recruitment through the release of materials [38].

It is time-intensive to develop a story with stakeholder input [15, 39], and it is less challenging ethically and practically to develop stories using research evidence alone [40, 41]. However, without community input, researchers risk overlooking important contextual factors in message development and delivery. Regardless of the degree to which users are involved in the modelling phase, the intervention team will be responsible for ensuring that the evidence-based messaging remains intact, and that messaging follows previously identified theory, mechanisms of action and BCTs.

#### Delivery medium

Stories can be delivered in numerous ways (e.g. printed storybook, video, and audio recording) and can include a variety of products, such as images, photos, drawings, voice-overs, or other visual and audio story vehicles. Regardless of the delivery format selected it must (1) be able to showcase the BCTs previously chosen to facilitate the mechanism of action and (2) be accessible by intended users. Researchers must consider contextual elements including who is delivering the message and channels of dissemination (e.g. waiting room TV, doctors, online, plays at events [20]); potential physical and cognitive disabilities, and literacy levels of the intended users (consider the pros and cons of text, video, audio messages); the mindset of the audience (e.g. readiness to change [42]), early vs. late adopters [19]; and how much time the user has to take in the messaging before making a decision or taking action. These types of distinctions in context highlight the value of co-creating the messaging tool with the knowledge users.

The available funds will affect the level of production and ability to create engagement [39]. One of the advantages of storytelling is that it can be cost-effective for researchers using formats such as simple text, PowerPoint presentations that include storytelling elements, or other formats that require little editing and printing resources. However, other resources will involve additional costs, such as editing software, web hosting, production specialists of any kind (involving expertise or equipment use), creative writers, visual artists, printing, or various forms of physical media.

#### Script development

Script development poses difficult tasks for those trying to use stories as KT interventions. How can we develop the problem and solution in the story in ways that make them impactful? How do we ensure that the knowledge user hears and remembers the message? And how do we ensure that in the story, our evidence-based messaging is not diluted or overshadowed by emotion or other techniques we use in the storytelling? To develop high-quality stories, the developers of the KT interventions must attend to (a) the form or forms of the stories to be developed and (b) the material that they intend to be the content of the stories.

### Script form

Stories are often described as formulaic (e.g. set the scene, establish the theme, present the plot, and come to a resolution) [43]. However, it is more accurate to describe this as the essential form of stories, as storytelling is a craft with no formula to create perfect messages that will resonate with users [44]. A story form can be involved, requiring many components. For example, a story can include an “abstract (what is the story going to be about),” “orientation,” “complicating action,” “evaluation,” “resolution,” and “coda” (or: what links the narration to the “present situation”) [45]. But an effective story can also be a single line, or even a single image, where an outcome may be inferred from a complication. Consider the John Caples single-line story, a classic among those who study advertising:

Complication: “They all laughed when I sat down at the piano…”

Resolution: “... but when I started to play!” (History of Advertising , n.d .).

The developers of a KT intervention will begin with a goal to modify behaviour (e.g. through persuasion, instruction, and information). This goal will ground the story in the form of the logic of a plot. Consider the one-line narration, “man takes swing at cat, loses balance, falls down stairs.” On its face, the logic is action-reaction, cause-and-effect, or ground-and-consequence. On another level, however, the logic that connects the actions could be irony, pratfall comedy, poetic justice, or a cautionary tale about safety around stairwells. The form of the story will differ considerably based on the related theory and behaviour change techniques. Similarly, the intervention theory will require decisions around types of narration (e.g. first, second, or third person; dramatized, or un-dramatized) [46], questions of showing (description) as opposed to telling (exposition), episodic organization, climactic organization, and so forth. For example, Banerjee and Greene (2012) tested various story conclusion formats for a story employing persuasive communication techniques to inspire people to decline offers to use cocaine [47]. They developed stories that depicted people who use cocaine either quitting cocaine and changing their life for the better (a positive, benefit-oriented ending) or suffering the consequences of ongoing cocaine use (a negative, cost-oriented ending). They found that positive endings resonated more with intended knowledge users. Both types of stories employ a persuasion BCT but creatively cultivate narratives that are completely different in form.

There is no formula to develop the perfect story-form for any particular situation. This is where the developers of KT interventions should rely on professionals with proven communications or marketing expertise. Hired professionals can help ensure that the form (sequence, content, plot, messaging traits, etc.) is high quality and memorable.

### Script material

The material of a story or series of stories can derive from either an informed imagination or lived experience. Stories developed from the informed imaginations come from those whose level of contact or experience allows them to generalize effectively, in collaboration with creatives whose task or profession is to develop effective stories. This means fiction, but this would be a fiction grounded in experience. Conversely, stories developed from lived experience require some type of participation from end-users or groups that influence them. These stories can take the form of testimonies, or generalizations derived from personal statements, interviews, etc. To ethically develop story material from lived experience, researchers must obtain permission and human ethics board reviews, where applicable, and spend time on due diligence to protect the safety, dignity, and anonymity of those who offer their time and their testimonies.

In any case, KT intervention developers must attend to the needs of their specific story audiences. Communications around climate change provide a salient example of audience complexities that must be considered during script development [48]. Stories meant to inspire action around climate change must account for audience beliefs, likelihood of accepting the information and changing behaviours, and likely reactions to various frames (e.g. messaging that conveys fear, motivation, support, and collaboration [48]). Again, including users in the script development can help bring their preferences and understandings to light, ensuring that the message is culturally appropriate and resonates with the intended audience. Some tensions between pre-existing beliefs can be addressed by acknowledging these beliefs but responding to them with the evidence-based messaging. For example, in story development for an asthma management project, Archibald et al. (2018) heard a common misconception that children can grow out of asthma. To emphasize the importance of the evidence-based asthma management recommendation, the team developed messaging that acknowledged common misconceptions while clarifying that asthma is a chronic (life-long), yet manageable, disease [28].

### Common challenges in script writing

While script writing can be an exciting activity from a creative point of view, researchers need to be cognizant of common challenges that arise at this stage of modelling. Maintaining evidence-based messaging can be difficult regardless of the narrative approach. There is often a tension between stories of extreme cases (which are common in the popular media but may cause more reaction and possibly fear) vs. stories of the typical case (which may engender less emotion but offer more comfort or reassurance). Further, the narratives may inadvertently contain inaccurate information about health outcomes, treatment, etc. [28], as in the asthma project described above [14, 28]. Teams must balance the desire for compelling stories (targeting memory, attention, and emotional resonance) with the need for delivering accurate, evidence-based messaging. To this end, teams can include professional storytelling facilitators to produce stories that are compelling and authentic, while involving content experts to ensure the messages are true to the scientific evidence.

Teams can mitigate problems of inaccurate messaging through rigorous story identification and selection processes that highlight compelling stories within an end-user population that connect directly with the intended KT messages, BCTs and mechanisms of action. In particular, when utilizing personal narratives, identifying the “right people” (e.g. purposeful sampling in qualitative research for articulate participants), and working with professional storytelling facilitators can produce stories that are authentic, compelling, and true to the evidence. However, in finding “the right people” teams may lose some generalizability by introducing stories that bring in inappropriate or alternative treatments/management approaches that are not supported by best evidence but that reflect reality (e.g. practice variation). Conversely, stories written with more specificity may be accurate but may not resonate with the target audience. In all cases, content experts should be involved in developing or at least reviewing the script for the accuracy of information/evidence presented.

#### Feasibility

Following the MRC guidance, teams should test or pilot their prototype before implementing the intervention to ensure its feasibility. Regardless of how the message is developed, this feasibility testing (also known as usability testing) is an important first step towards ensuring that the storytelling intervention will have the intended behaviour change effects and address the modelling challenges described above. Otherwise, researchers risk both planning and messaging pitfalls that may cause the intervention to fail [8].

As with message and delivery development, feasibility testing relies on input from the end-user audience. For example, the audience can inform the tension between compelling and factually correct messaging described in the last section. Their input is important for identifying whether a story is or is not compelling enough to achieve its purpose while maintaining its evidence-based message. In addition, to test the story itself, end-users can provide input on the impact of message delivery aspects, such as video elements, drawings, and voice-overs.

Feasibility measurements can be evaluated in a number of ways and can be revisited as the intervention is scaled up or tailored for new audiences. For settings and interventions that cannot directly measure behaviour changes or health outcomes, surrogate measurements have been established to help assess whether the use of storytelling is moving people towards the intended behaviour. Such measurements include transportation, homophily, realism, and recall. Transportation, or the level of user engagement with the message, is measured using the transportation scale [49]. A related measure, homophily, is the degree of perceived similarity between characters and the audience. Increasing homophily will increase the ability of the message user to engage with the message (transportation). Homophily can be measured using the perceived homophily in the interpersonal communication scale [50].

Increasing realism is another pathway to improving the likelihood of transportation. Realism (whether the story is perceived as authentic or similar to real life) can be measured using the perceived plausibility subscale of the perceived reality scale [51]. Useful evidence in storytelling literature showed that if a team uses personal narratives from target community members in the target setting, they can assume homophily and realism [15, 52]. One challenge identified with this method is that incorporating research evidence into personal narratives can reduce realism as the evidence may be new or contrary to prior beliefs [14, 28]. Using creative solutions, researchers can acknowledge personal experiences and introduce new evidence [14], in turn achieving desired transportation, homophily, and realism.

Together transportation, homophily, and realism contribute to user recall: the ability to remember and relay information accurately. Recall is measured by asking people what they remember about the story [53]. Recall is a helpful measure to evaluate the script development and whether the story is understandable and memorable. However, considering storytelling as a complex intervention, teams use available measurements to evaluate all the various intervention components, such as delivery method, timing, and dose.

### Evaluation phase

After modelling, pilot testing, and implementing the storytelling intervention, the MRC Framework suggests evaluating the intervention. When using a method like storytelling, that involves significant effort in building the story, one risks focusing on evaluation of the story alone (e.g. focusing on feasibility measurements described above). In the MRC guidance, evaluation comprises: (1) assessing effectiveness, (2) understanding change process, and (3) assessing cost-effectiveness. This calls for multiple and/or mixed methods for evaluation. To assess effectiveness, researchers must carefully select outcomes, measures, and timing of assessments that will reflect whether the intervention achieved its intended purpose [14, 54]. Researchers should reflect on the theory stage and the means by which the intervention can change behaviour, then select outcomes reflective of their aims (e.g. increase knowledge, change attitudes, create intentions, change behaviour, and improve health outcomes). Using and evaluating BCTs here can help measure the link between purpose and actual change in behaviour. Depending on the design and timelines for the evaluation, researchers may need to rely on more proximal outcomes (e.g. knowledge, attitudes, intentions) to reflect the possibility of achieving more distal effects (e.g. behaviour change, improved health outcomes). Some frameworks exist for conceptualizing effects/outcomes [55–57] for instance, Coulter and Ellins group outcomes/effects into patient knowledge, patient experience, health behaviour and health status, and service utilization and costs (Table 4) [58]. This example shows how establishing the theoretical basis for the intervention in the initial stage can make this step more straightforward.

**Table 4:** Outcomes for assessing patient-focused interventions [58]

Outcome category	Examples
Patients’ knowledge	● Knowledge of condition and long term complications ● Self-care knowledge ● Knowledge of treatment options and likely outcomes ● Comprehension of information ● Recall of information
Patients’ experience	● Patient satisfaction ● Doctor-patient communication ● Quality of life ● Psychological wellbeing ● Self-efficacy ● Patient involvement
Service utilization and costs	● Hospital admissions ● Emergency admissions ● Length of hospital stay ● GP visits ● Cost-effectiveness ● Cost to patients ● Days lost from work/school
Health behaviour and health status	● Self-care activities ● Treatment adherence ● Disease severity/activity ● Symptom control ● Functional ability ● Clinical indicators

Drawing on recommendations for KT interventions, evaluations should focus on internal validity, that is “the degree to which an observed outcome can be attributed to an intervention” [59]. Randomized controlled trials provide the most robust evidence (with practical options if a conventional parallel design is not possible [8]), though non-randomized designs (e.g. uncontrolled before and after, controlled before and after, interrupted time series) or observational studies (with adequate adjustment for confounding) may be easier to implement [8, 59]. To gain insight into the change process (e.g. reasons the intervention does or does not achieve its intended purpose), qualitative methods should accompany the effectiveness evaluation [59]. Finally, consideration of health economics as a component of the evaluation is important to assess the impact (outcomes) given the costs of developing and implementing the intervention [60].

These considerations for evaluation are drawn from fields that have implications for health [8] and KT in health care [61]. Authors across disciplines who use storytelling acknowledge the number of theoretical perspectives available to inform story development [1]. These different perspectives pose challenges for storytelling interventions. Indeed, the storytelling framework presented in this article integrates TDF, COM-B, and the MRC Framework in an effort to provide guidance that is relevant across perspectives. While health research approaches are often informed by the post-positivist philosophical tradition [1], storytelling as a method follows more subjective social science and arts-based traditions to identify any intervention impacts. There have been calls for the development of a unified narrative theory or formal pedagogy [62, 63]; however, until such a pedagogy emerges, researchers can rely on mixed-methods approaches that evaluate both the science and the art in storytelling interventions.

## Discussion

This framework helps researchers consider several important elements when developing KT interventions that include stories and there are key threads that run throughout the theory, modelling and evaluation phases. As with other arts-based KT, developing effective stories in KT requires stakeholder involvement [64]. Involving stakeholders in story development is ideal for creating stories that are relevant and impactful for the intended audience [14]. For example, Houston et al. (2011) chose oral storytelling to reach their African American smoker population as oral stories are deeply entrenched in African American culture [15]. To develop effective messages, they recorded members from their target audience speaking about their experiences with smoking and smoking cessation. In this way, they could choose stories that mapped to BCTs and that used language and examples that resonated with the intended audience [15]. Our scan of the literature focused on reports of stories in and of themselves, separate from their relationship to different storytelling media. Further framework development can inform how to best involve stakeholders in story development for different story mediums (e.g. text, theatre, visual art, and music).

The storytelling literature uncovered gaps in reporting both the theory underpinning stories and the measures used to evaluate stories in relation to the intervention goals [6, 7]. Rigorous, theory-based KT requires researchers to understand and build their interventions around the goals for change [15]. However, the theory of change is underreported in the current storytelling literature. Furthermore, existing literature stated the utility of quantitative measures (e.g. transportation, homophily, realism, and recall [47, 48];) accompanied by qualitative evaluation data [59], but the limited reporting of evaluation processes and results limit the understanding of how to use and tailor these measures for different storytelling formats and mediums. In line with findings of evaluation in other arts-based KT, reported evaluations focused mainly on immediate impact [64]. The KT research community would benefit from explicit articulations of intervention theory and subsequent evaluations as these efforts will help researchers develop theory-based KT interventions that incorporate stories effectively. The storytelling framework developed here provides a structure to report lessons learned in intervention development from theory through modelling, feasibility testing, and evaluation.

In developing the framework, we drew on best practices for developing and using stories from a wide range of disciplines and we involved an interdisciplinary team of experts. Furthermore, we grounded the framework with behaviour change theory and the well-established MRC Framework [16] for developing and evaluating complex interventions. However, there is debate around whether the theoretical constructs presented support both individual as well as group (e.g. family) decision-making and action. Additionally, given that storytelling is used across research traditions, the applicability of this framework may be better suited for some approaches while others may need different guidance than what is provided here. Future work to test and refine this framework will be valuable; the author team of this article welcomes feedback from researchers who have used it.

## Conclusion

This framework is a first effort towards developing practical guidance for designing story-based KT interventions. The intention of the framework is to present considerations for storytelling, as opposed to being prescriptive. We used a complex intervention lens paired with existing BCTs to guide appropriate theory-based intervention planning and practical choices. An intentional approach to the development of story-based KT interventions should involve three phases. The theory phase specifies the goal of the intervention, mechanisms of action, and behaviour change techniques that will achieve the intended effects. The modelling phase involves development and testing using an iterative approach, multiple methods and involvement of end-users. Finally, formal evaluation using multiple methods helps determine whether the intervention is having its intended effects and value added. The framework also offers an opportunity to further develop theory and the community’s understandings of mechanisms of action in storytelling interventions.

## Supplementary Information

**Additional file 1.**

## Data Availability

Data collected to inform the study is based on previously published papers; a list of papers is provided in Addition file 1.

## Abbreviations

APEASE: Affordability, practicality, effectiveness/cost-effectiveness, acceptability, side-effects/safety, equity
BCT: Behaviour change techniques
BCW: Behaviour Change Wheel
COM-B: Capability, Opportunity, Motivation to Behaviour
KT: Knowledge translation
MRC: Medical Research Council
