# Supplementary material for: A framework to guide storytelling as a knowledge translation intervention for health-promoting behaviour change
Source: Implement Sci Commun. 2022 Mar 28;3:35. doi: 10.1186/s43058-022-00282-6 (PMC8962242; doi:10.1186/s43058-022-00282-6)
Supplement: Supplementary file 1 — Additional file 1. [file 43058_2022_282_MOESM1_ESM.docx]

**Additional file 1 Information**

File name: Additional file 1

File format: Word document (.docx)

Title of data: List of Storytelling Literature Collected

Description of data: literature used to build framework presented in this manuscript

**Additional file 1: List of Storytelling Literature Cited**

1. Alberta Addiction & Mental Health Research Partnership Program. Creative KT: Ideas and Resources. 2015. Edmonton, AB: Alberta Health Services. <https://www.albertahealthservices.ca/assets/info/res/mhr/if-res-mhr-creative-kt.pdf>. Accessed 2 Sept 2021.
2. Alberta Innovates - Health Solutions. Bridging the Gap - Knowledge Translation in Alberta KT Casebook. Alberta Innovates-Health Solutions. 2010. <https://fliphtml5.com/nums/dgtc>. Accessed 2 Sept 2021.
3. Banerjee SC, Greene K. 'I quit' versus 'I'm sorry I used': a preliminary investigation of variations in narrative ending and transportation. Psychol Health. 2012;27(11):1308-22.
4. Bekker HL, Winterbottom AE, Butow P, Dillard AJ, Feldman-Stewart D, Fowler FJ, Jibaja-Weiss ML, Shaffer VA, Volk RJ. Do personal stories make patient decision aids more effective? A critical review of theory and evidence. BMC Med Inform Decis Mak. 2013;13 Suppl 2:S9.
5. Boeijinga A, Hoeken H, Sanders J. Storybridging: Four steps for constructing effective health narratives. Health Educ J. 2017;76(8):923-935.
6. Cameron KA. A practitioner's guide to persuasion: an overview of 15 selected persuasion theories, models and frameworks. Patient Educ Couns. 2009;74(3):309-17.
7. Couldry N. Digital storytelling, media research and democracy: conceptual choices and alternative futures.In: Lundby, Knut, editor. Digital storytelling, mediatized stories: self-representations in new media. New York, NY, USA: Peter Lang Publishing, Inc. 2008. p. 41-60.
8. Downs JS, Murray PJ, Bruine de Bruin W, Penrose J, Palmgren C, Fischhoff B. Interactive video behavioral intervention to reduce adolescent females' STD risk: a randomized controlled trial. Soc Sci Med. 2004t;59(8):1561-72.
9. Dunlop SM, Kashima Y, Wakefield M. Predictors and consequences of conversations about health promoting media messages. Commun Monogr. 2010;77(4):518-39.
10. Dunlop SM, Wakefield M, Kashima Y. Pathways to persuasion: Cognitive and experiential responses to health-promoting mass media messages. Comm Res. 2010;37(1):133-64.
11. Ford N, Koetsawang S. A pragmatic intervention to promote condom use by female sex workers in Thailand. B World Health Organ. 1999;77(11):888.
12. Gallagher KM. In search of a theoretical basis for storytelling in education research: Story as method. Int J Res Method Educ. 2011 Apr 1;34(1):49-61.
13. Goddu AP, Raffel KE, Peek ME. A story of change: The influence of narrative on African-Americans with diabetes. Patient Educ Couns. 2015;98(8):1017-24.
14. Greene K, Brinn LS. Messages influencing college women's tanning bed use: Statistical versus narrative evidence format and a self-assessment to increase perceived susceptibility. J Health Comm. 2003;8(5):443-61.
15. Greene K, Campo S, Banerjee SC. Comparing normative, anecdotal, and statistical risk evidence to discourage tanning bed use. Comm Quart. 2010;58(2):111-32.
16. Greenhalgh T, Hurwitz B. Why study narrative? BMJ. 1999;318(7175):48-50.
17. Gucciardi E, Jean-Pierre N, Karam G, Sidani S. Designing and delivering facilitated storytelling interventions for chronic disease self-management: a scoping review. BMC Health Serv Res. 2016;16:249.
18. Hernandez MY, Organista KC. Entertainment-education? A fotonovela? A new strategy to improve depression literacy and help-seeking behaviors in at-risk immigrant Latinas. Am J Community Psychol. 2013;52(3-4):224-35.
19. Hopfer S, Garcia S, Duong HT, Russo JA, Tanjasiri SP. A narrative engagement framework to understand HPV vaccination among Latina and Vietnamese women in a planned parenthood setting. Health Educ Behav. 2017;44(5):738-47.
20. Houston TK, Cherrington A, Coley HL, Robinson KM, Trobaugh JA, Williams JH, Foster PH, Ford DE, Gerber BS, Shewchuk RM, Allison JJ. The art and science of patient storytelling—harnessing narrative communication for behavioral interventions: the ACCE project. J Health Comm. 2011;16(7):686-97.
21. Lewis PJ. Storytelling as research/research as storytelling. Qual Inq. 2011;17(6):505-10.
22. Mello R. The Power of Storytelling: How Oral Narrative Influences Children's Relationships in Classrooms. Int J Educ Arts. 2001;2(1).
23. Murphy ST, Frank LB, Chatterjee JS, Moran MB, Zhao N, Amezola de Herrera P, Baezconde-Garbanati LA. Comparing the Relative Efficacy of Narrative vs Nonnarrative Health Messages in Reducing Health Disparities Using a Randomized Trial. Am J Public Health. 2015;105(10):2117-23.
24. Nguyen HL, Ha DA, Goldberg RJ, Kiefe CI, Chiriboga G, Ly HN, Nguyen CK, Phan NT, Vu NC, Nguyen QP, Allison JJ. Culturally adaptive storytelling intervention versus didactic intervention to improve hypertension control in Vietnam- 12 month follow up results: A cluster randomized controlled feasibility trial. PLoS One. 2018; 31;13(12):e0209912.
25. Perrier MJ, Martin Ginis KA. Changing health-promoting behaviours through narrative interventions: A systematic review. J Health Psychol. 2018;23(11):1499-1517.
26. Perrier MJ, Martin Ginis KA. Narrative interventions for health screening behaviours: A systematic review. Journal of Health Psychology. 2017;22(3):375-93.
27. Plaisant C, Druin A, Lathan C, Dakhane K, Edwards K, Vice JM, Montemayor J. A storytelling robot for pediatric rehabilitation. In: Proceedings of the fourth international ACM conference on Assistive technologies. 2000:50-55. Doi: 10.1145/354324.354338
28. Price S. Knowledge translation for a new generation. Canadian Nurse. 2016. <https://canadian-nurse.com/en/articles/issues/2016/november-2016/knowledge-translation-for-a-new-generation>. Accessed 2 Sept 2021.
29. Reid K, Hartling L, Ali S, Le A, Norris A, Scott SD. Development and usability evaluation of an art and narrative-based knowledge translation tool for parents with a child with pediatric chronic pain: multi-method study. J Med Internet Res. 2017;19(12):e8877.
30. Robin B. The educational uses of digital storytelling. In C. Crawford, R. Carlsen, K. McFerrin, J. Price, R. Weber & D. Willis (Eds.), Proceedings of SITE 2006--Society for Information Technology & Teacher Education International Conference (pp. 709-716). Orlando, Florida, USA: Association for the Advancement of Computing in Education (AACE). https://www.learntechlib.org/primary/p/22129/. Accessed 2 Sept 2, 2021.
31. Sadik A. Digital storytelling: A meaningful technology-integrated approach for engaged student learning. Educ Tech Res Dev. 2008;56(4):487-506.
32. Shelby A, Ernst K. Story and science: how providers and parents can utilize storytelling to combat anti-vaccine misinformation. Hum Vacc Immunother. 2013;9(8):1795-801.
33. Smith B, Tomasone JR, Latimer-Cheung AE, Martin Ginis KA. Narrative as a knowledge translation tool for facilitating impact: Translating physical activity knowledge to disabled people and health professionals. Health Psychol. 2015;34(4):303.
34. Solórzano DG, Yosso TJ. Critical race methodology: Counter-storytelling as an analytical framework for education research. Qual Inq. 2002;8(1):23-44.
35. Sundin A, Andersson K, Watt R. Rethinking communication: integrating storytelling for increased stakeholder engagement in environmental evidence synthesis. Environ Evid. 2018;7(1):1-6.
36. Wieland ML, Njeru JW, Hanza MM, Boehm DH, Singh D, Yawn BP, Patten CA, Clark MM, Weis JA, Osman A, Goodson M. Pilot feasibility study of a digital storytelling intervention for immigrant and refugee adults with diabetes. Diabetes Educator. 2017;43(4):349-59.
37. Woodside AG. Brand‐consumer storytelling theory and research: Introduction to a Psychology & Marketing special issue. Psychol Marketing. 2010;27(6): 531-40.
38. Wolitski RJ, Fishbein M, Higgins DL, Rietmeijer C, Guenther-Grey CA, Johnson WD. Community-level HIV intervention in 5 cities: final outcome data from the CDC AIDS Community Demonstration Projects. Am J Public Health. 1999;89(3):336-45.
39. Zhou Z, Cheok AD, Pan J, Li Y. Magic Story Cube: an interactive tangible interface for storytelling. In: Proceedings of the 2004 ACM SIGCHI International conference on advances in computer entertainment technology. 2004.
